# Inhibitory studies of HepG2 and human immune cells using Solanum nigrum

**DOI:** 10.6026/97320630016525

**Published:** 2020-07-31

**Authors:** Anbazhagan Ganesh Kumar, Giridharan Bupesh, Palanandi Sankarganesh, Sakthivel Vasanth, Siddan Nandagopal, Baby Joseph

**Affiliations:** 1 Department of Microbiology, Hindustan College of Arts and Science, Padur, Chennai - 603 103, Tamil Nadu, India; 2 Center for Research and Consultancy, Hindustan Institute of Technology and Science, Padur, Chennai-603103 Tamil Nadu, India; 3 Research and Development Wing, Sree Balaji Medical College and Hospital, Bharath Institute of Higher Education and Research, Chromepet - 600 042, Tamil Nadu, India; 4 Department of Botany, Government Arts College, Hosur 635001, Tamil Nadu, India; 5 Department of Forest Science, School of Science, Nagaland University (Central), Lumami, Zenheboto, Nagaland-798627, India

## Abstract

It is of interest to document the inhibitory studies of HepG2 and human immune cells using Solanum nigrum. We have assessed the effect of ethanolic extract from Solanum nigrum for antiproliferative on HepG2 and on human PBMC. Treatment with Solanum nigrum separates the cells that are estimated by 3-[4,5-dimethylthiazol-2-yl]-2,5-diphenyl tetrazolium bromide measure. Results indicated improved cytotoxicity with expanding concentrations of extract.

## Background

The Indian subcontinent is a home to 16.5% of the total populace and it is evaluated that there are more than 2 million individuals with malignant growth. Right now in India, out of a million recently analyzed malignant growth patients consistently and another a million disease survivors show progressive infections within 5 years of analysis. In 2005, cancer killed around 8.26 lakhs individuals in India; 5.19 lakhs under the age of seventy. This is predictable to rise disproportionally in correlation with cardiovascular and transferable diseases by means of 2030, to about 1.5 million deaths annual. In view of the most cancers arithmetic information it's far expected that there might be around 800,000 new tumors occasions in India every year [1].

The immune system is complex organ high specific cells, which are protecting the body from the contamination. Innate immunity mostly depends upon granulocytes and macrophages, even as adaptive immune reaction relies on lymphocytes, which give long expression immunity. Immunomodulation is the choice of immune reactions by avoid them to forestall transmittable diseases or through smothering them inside the undesired conditions. Natural compounds such as proteins, amino acids have a ability adjust immune responses, alongside interferon-γ (IFN- γ),steroids. Plants were used considering that historical instance for the remedy of numerous diseases and issues [2,3].

Immunodeficiencies occur while at least one of the segments the immune system is inactive. It incorporated hypersensitivity, autoimmunity, HIV and so on [4- 6]. New immunomodulatory plants are significant for the finding of drug with fewer symptoms, low expensive, extra powerful and valuable treatment developed for immune and their associated diseases. Natural drugs are open from side effects and toxic quality not at all like to the allopathic drugs.

The historical backdrop of plant as wellspring of enemies of cancer-causing agents started in the soonest 1950s, with the establishment and improvement of the vinca alkaloids (vinblastine and vincristine) and the separation of the cytotoxic podophyllotoxins. Vinca alkaloid is answerable for enhance in the rates for Hodgkin’s issue and couple a multiplicity of leukemia. Vincristine inhibits microtubule gathering, inducing tubulin self-association into coiled spiral aggregates.

Etoposide is an epipodophyllotoxin, got from the mandrake plant Podophyllum peltatum and the wild chervil Podophyllum emodi. It has additionally huge importance towards small-cell lung carcinoma. Etoposide is a topoisomerase II inhibitor, stabilizing enzyme-DNA cleavable complexes leading to DNA breaks. The taxanes i.e., paclitaxel and docetaxel has been show antitumor action in breast, ovarian and distinctive tumor sorts in the emergency clinic preliminary. In the present study the role of Solanum nigrum as an inhibitor of cancerous cell and its antiproliferative effect on HepG2 and human immune cells was assessed.

## Materials and Methods:

### Collection and processing of the plants:

Healthy was collected and recognized by an Indian anatomical plant research center, Chennai. Plant was washed with running tap water and was shade dried. Care was taken to stay away from infectious tainting while at the same time drying. The dried plant was stored and proceeds for homogenization further use.

### Preparation of the extract-cold extraction:

The Solanum nigrum leaves were powdered utilizing mechanical blender. 100gms of powder was extracted with 200 ml of ethanol and then concentrated.

### Cytotoxicity study:

The cytotoxic effects of various concentrations of Solanum nigrum extracts were analysed on PBMC by performing three tests Viz. Color rejection technique, Sulforhodamine B test and MTT.

### Clonogenic assay:

The impact of treatment on clonogenic survival of disease cells was resolved utilizing colony formation assay. The cells were treated with expanding focuses (20, 40 80 and 160µg/ml) of the plant extricates in RPMI-1640 complete medium. Following treatment, the cells were re-plated in triplicate on a 6-well tissue culture plate with 5000 cells/well and refined in 5% CO2 at 37°C for 8 days with development media being supplanted with/without removes at regular intervals. The cells were then stained with 0.5% gem violet (in methanol: H2O; 1:1) and the outcomes were noted.

### Effect of the extracts and the expression of Bcl-2 protein by Western blotting on HepG2 cells:

Cell incubated and medication organization: HepG2 cells taken in the phase of logarithmic development and were processed with pancreatin, and the cell concentrationwas balanced by addingRPMI1640 culture medium with 10% fetal ox-like serum. The example was then placed in Petri dishes at different focus in dish and incubated in CO2 incubator for 24 h to cause the cells to hold fast to the inward mass of the dishes. The examination was performed on a negative control, a positive control, and with various concentrates at different extracts of 20, 40, 80 and 160 µg. The examples were then incubated in the CO2 incubator at 5% CO2 and 37°C for 48 h.

### Preparation of the examples:

One ml of PBS was added to the way of life jar. The cells were scratched utilizing a cell scrubber, added to a 1.5 ml axis tube, and centrifuged at 1500 rpm for 5 min, after which the example was washed twice with PBS. One hundred microlitre of cell lysatewas added to the example, whichwas then spun, after which the example was lysed from 30 min in a frosted waterbath. It was then centrifuged for 10 min at 4°C and 12,000xg. The supernatant was gathered and refrigerated at −20°C for later use. Assurance of the protein content was finished by Bradford technique (Kruger, 1994) to decide the protein content.

### Electrophoresis:

Twelve percent resolving Gel and five percent Stacking Gel were prepared. Examples were taken from the examples as per different protein substance, SDS buffer solutiont was added and blended in with the examples, after which the examples were boiled for 5 min in 100°C water bath. The examples were mounted in the wake of being chilled off. The gel was run at a voltage of 80V, which was then changed to 110V after the examples had arrived at the partition gel.

### Transfer:

Following electrophoresis, the gel was stripped and cut off, and afterward moved for 2 h at 50 mA. The film was taken out, fixed with TTBS containing 3% BSA, and afterward set under 4°C for the evening. The layer was flushed with TTBS multiple times for 5 min/time, after which murine enemy of human Bcl-2 immune response I weakened 1:250 was included. After three hours, the film was again washed with TTBS multiple times for 5 min/time, after which hostile to murine IgG counter acting agent II weakened 1:250 and marked with antacid phosphatase was included. Beta-actin was utilized as control. The film was taken out after 2 h and flushed multiple times with TBS, 5 min each time. Film was then created utilizing western blot discovery reagent.

### Quantification of IL-2:

IL-2 some time ago called as development factor, is a 14 - 16 kDA glycosylated polypeptide created by activated CD4+ Th cells which acts inside an autocrine approach to advance T cells and NK cells development. Lymphocytes react to IL-2 by means of official to the high proclivity IL-2 receptor made up of three subunits (α, β, γ). IL-2 can be found in culture supernatant after mitogen initiation of mononuclear cells (PBMC) or T cell clones.

Different concentration of the SN extract were arranged and subjected for the in-vitro examination on human PBMC alone treated with mitogen to evaluate IL-2. Consistent cell number was kept up. The test was performed in a 96 well tissue culture plate (Greiner, U.S.A), utilizing different negative controls like plain media, complete media, vehicle, cell and concentrate. Positive controls like a known immunomodulator (PHA) and a known cytotoxic compound (LPS) were likewise kept up. To measure IL-2 discharged mitogen enacted human PBMC were treated with the concentrate. After the expansion of cell, media, mitogen and concentrate, the way of life were hatched in a hatchery (TC2323, Shel lab, U.S.A) with 95% air, 5% CO2 and humidified environment at 37°C for 72 hrs. After the hatching time frame, the way of life supernatant was taken and utilized for measurement of IL-2 emitted. IL-2 was evaluated by utilizing IL-2 ELISA unit made by M/s. Bio Source, Belgium.

### Statistical analysis:

Statistical analysis was finished utilizing EXCEL (DATA ANALYSIS) for deciding Standard error and Standard deviation. A p estimation of < 0.01 was considered factually exceptionally noteworthy, p < 0.05 critical and p 7gt; 0.05 in significant.

## Results:

### Immunomodulatory and cytotoxic nature of the extracts:

**Figure 1:**
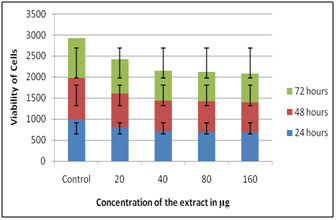
DEM on PBMC using Solanum nigrum extracts

**Figure 2:**
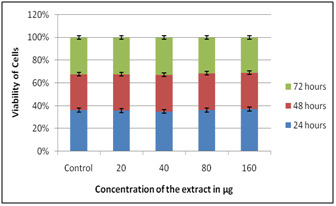
DEM on HepG2 using Solanum nigrum extracts

**Figure 3:**
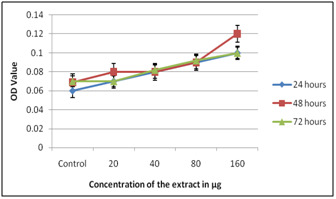
SRB on PBMC using Solanum nigrum extracts

**Figure 4:**
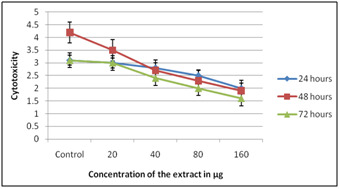
SRB on HepG2 using Solanum nigrum extracts

When DEM was performed utilizing Solanum nigrum extract it was seen as non-toxic on all concentration when presented to 72 hours. While cell multiplication (i.e.) immunomodulatory impact was found at 80 µg when presented to 72 hours (Figure 1,Figure 2). On HepG2 it was seen as non- toxic on all concentration when presented to 72 hours. Results dependent on SRB demonstrated that both on PBMC and on HepG2 the concentrates expanded the expansion of cells clear by expanded in the OD esteem. When MTT test was performed on PBMC the concentrates expanded the expansion of cells comparative outcomes were gotten on treatment with HepG2 (Figure 3, Figure 4) and (Table 1).

### Clonogenic assay and Western Blotting:

**Figure 5:**
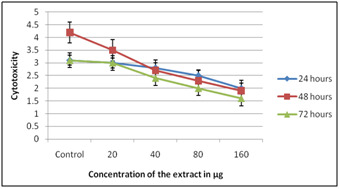
MTT on PBMC using Solanum nigrum extracts

**Figure 6:**
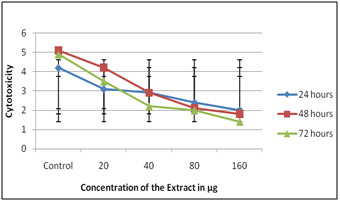
MTT on HepG2 using Solanum nigrum extracts

Clonogenic examine refers to the capacity of the cell to multiply and holds its ordinary morphological trademark when the plant extacts were added to the cancer cells. The loss of reproducible capacity and powerlessness to multiply is the normal reason for the cell death, which was clear after a time of concentrate presentation. Results revealed that the concentrate has the antiproliferative action on Hep G2 cell line. Western blot examination appears, that with the expansion in the measurements of the plant extricate and the substance of Bcl-2 protein in HepG2 cells decreased (Figure 5,Figure 6).

### Interleukin 2 (IL2):

**Figure 7:**
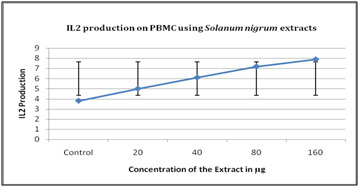
IL2 production on PBMC using Solanum nigrum extracts

Human PBMC alone when treated with the plant extricate which delivered great amount of IL2 at expanded Concentration. There was an immediate connection between's concentration of the extract and the quantity of IL2 delivered (Figure 7).

## Discussion:

Many scientific data have established the pharmacological results of steam distilled, petroleum ether, benzene extracts of numerous elements of plant and its active ingredient eugenol on diverse systems like immune system, reproductive system, cardiovascular system, gastric system, and blood. In current years, ethnobotanical and conventional makes use of of herbal compounds, particularly plant origins plays a vital role. They deserve scrutiny on present day medical strains such as physiochemical characterization, organic assessment, toxicity research, research of molecular mechanism of action(s) of isolated phytoprinciple and their medical trials [7-9]. These are necessary classical processes in seek of new lead molecule for management of diverse diseases. Enhancement of antigen presentation to T cellular is crucial immunomodulatory assets of any drug/extract. In a study, extracts were tested; maximum enhancement was located in E. Nummularius treated antigen presenting cells. A tumor is a disease characterised with the aid of a proliferation disorder and an apoptosis obstacle. Inducing apoptosis is an effective method of treating cancers. Investigations became initiated to study the anticancer potential of Methanolic extract of Parkia javanica (MEPJ) and Methanolic extract of Evolvulus nummularius (MEEN) in vitro against exclusive cancer cellular traces (such as conventional drug resistant cancer line). The study shows that the extracts were powerful in providing increase inhibition in-vitro towards diverse human most cancers cell lines (such as traditional drug resistant lymphoblastic leukemia). Dose based response was discovered. The increase inhibitory impact of MEPJ becomes extra outstanding in opposition to K562 and CHO [10-12]. One of the significant speculations related with disease chemoprevention and phytochemicals shows that high cell reinforcement limit is corresponded with a high centralization of phytochemicals and in this way with progressively anticancer action [13]. Recently, the antiproliferative effect of S. nigrum has also been shown against various cancer cell lines. The antitumour impact of SNE has been demonstrated because of the presence of polysaccharide, glycoalkaloids or glycoproteins [14]. The polysaccharides have been appeared to apply antiproliferative impact due to their immunomodulatory properties while glycoalkaloids and glycoproteins have been appeared to activate the proapoptotic factors or inhibit the transcription factors playing an important role in tumour progression. In this study TLC of the plant extracts showed steroids, alkaloids and tannins. It has been reported recently that traditional uses of natural compounds, especially of natural origin received much attention as they are well tested for their efficacy and generally believed to be safe for treating mankind. Herbal medicine deserves study on recent scientific outline such as physiochemical characterization, biological evaluation, toxicity studies, study of molecular system of action and their clinical trials [15, 16]. It seems that these are necessary classical approaches in search of new lead molecule for management of various diseases. In the future the isolated principles of the plants chosen needs to be evaluated in scientific manner using various innovative experimental models and clinical trials to understand its mechanism of action, in search of other active constituents, so that its other therapeutic uses can be widely explored which can benefit mankind not only in curing dreadful disease like cancer but for many such diseases.

## Conclusions:

The ethanolic extract of S. nigrum was found to have high cytotoxic effect on the liver cancer cell line HepG2. The extract enhances the proliferation of PBMC when compared with HepG2cells. Thus the S. nigrum is a potent antitumor agent in the context of liver cancer.

## Figures and Tables

**Table 1:** Results of Clonogenic assay on HepG2 Cell lines treated with Solanum nigrum

S. No	Plant Extracts	Concentration of the extract in µg	Time Exposure		
			24 hours	48 hours	72 hours
1	Solanum nigrum	20 µg	NT	NT	AP
		40 µg	NT	NT	AP
		80 µg	NT	AP	AP
		160 µg	NT	AP	AP
NT-Non Toxic/Normal;AP-Antiproliferative
